# Deciphering the molecular landscape: integrating single-cell transcriptomics to unravel myofibroblast dynamics and therapeutic targets in clear cell renal cell carcinomas

**DOI:** 10.3389/fimmu.2024.1374931

**Published:** 2024-03-18

**Authors:** Wenqian Zhou, Zhiheng Lin, Wang Tan

**Affiliations:** ^1^ Tongji Hospital, School of Medicine, Tongji University, Shanghai, China; ^2^ Shandong University of Traditional Chinese Medicine, Jinan, Shandong, China; ^3^ Xiangya Boai Rehabilitation Hospital, Changsha, Hunan, China

**Keywords:** HMGA1+ myofibroblasts, ccRCCs, ScRNA-seq, bulk RNA-seq, experiment validation

## Abstract

**Background:**

Clear cell renal cell carcinomas (ccRCCs) epitomize the most formidable clinical subtype among renal neoplasms. While the impact of tumor-associated fibroblasts on ccRCC progression is duly acknowledged, a paucity of literature exists elucidating the intricate mechanisms and signaling pathways operative at the individual cellular level.

**Methods:**

Employing single-cell transcriptomic analysis, we meticulously curated UMAP profiles spanning substantial ccRCC populations, delving into the composition and intrinsic signaling pathways of these cohorts. Additionally, Myofibroblasts were fastidiously categorized into discrete subpopulations, with a thorough elucidation of the temporal trajectory relationships between these subpopulations. We further probed the cellular interaction pathways connecting pivotal subpopulations with tumors. Our endeavor also encompassed the identification of prognostic genes associated with these subpopulations through Bulk RNA-seq, subsequently validated through empirical experimentation.

**Results:**

A notable escalation in the nFeature and nCount of Myofibroblasts and EPCs within ccRCCs was observed, notably enriched in oxidation-related pathways. This phenomenon is postulated to be closely associated with the heightened metabolic activities of Myofibroblasts and EPCs. The Myofibroblasts subpopulation, denoted as C3 HMGA1+ Myofibroblasts, emerges as a pivotal subset, displaying low differentiation and positioning itself at the terminal point of the temporal trajectory. Intriguingly, these cells exhibit a high degree of interaction with tumor cells through the MPZ signaling pathway network, suggesting that Myofibroblasts may facilitate tumor progression via this pathway. Prognostic genes associated with C3 were identified, among which TUBB3 is implicated in potential resistance to tumor recurrence. Finally, experimental validation revealed that the knockout of the key gene within the MPZ pathway, MPZL1, can inhibit tumor activity, proliferation, invasion, and migration capabilities.

**Conclusion:**

This investigation delves into the intricate mechanisms and interaction pathways between Myofibroblasts and ccRCCs at the single-cell level. We propose that targeting MPZL1 and the oxidative phosphorylation pathway could serve as potential key targets for treating the progression and recurrence of ccRCC. This discovery paves the way for new directions in the treatment and prognosis diagnosis of ccRCC in the future.

## Introduction

Renal neoplasia stands as one of the most prevalent genitourinary malignancies globally (1). According to data disseminated by the World Health Organization, the annual mortality toll attributed to renal cancer reaches a staggering 140,000-170,000 patients (2, 3). Among these cases, clear cell renal cell carcinomas (ccRCCs) emerge as the predominant histological subtype, contributing to approximately 80% of renal cancers and representing the clinical variant with the highest mortality rates (3–5). Presently, localized instances of ccRCCs are conventionally addressed through surgical resection (6, 7). However, due to the latent nature of symptoms during the early stages and the proclivity for tumor metastasis by the time of detection, reliance solely on surgical intervention becomes less efficacious (8). Distinguishing ccRCCs from other genitourinary tumors lies in their refractoriness to chemotherapy and radiotherapy (9). The prevailing use of chemotherapy and cytokine therapy involving interferon-alpha (INFα) and IL-2 not only presents challenges in ensuring efficacy but also manifests susceptibility to drug resistance and adverse effects (10). Furthermore, the overall survival rate for advanced ccRCCs languishes below 30% (11, 12). Consequently, an imperative exists to investigate the fundamental mechanisms and signaling pathways governing the progression and recurrence of ccRCCs, identify novel therapeutic targets, and formulate innovative prognostic models.

Cancer-associated fibroblasts (CAFs) are activated fibroblasts found within or in the vicinity of cancerous tissues, closely associated with cancer progression. Currently, it is speculated that they primarily originate from resident fibroblasts and can modulate certain cellular mechanisms of tumors, including functions related to tumor proliferation, invasion, and metastasis, leading to adverse prognoses in affected patients (13–17). Existing research has revealed that fibroblasts can promote the tumor drug resistance of ccRCCs through the TDO/Kyn/AhR signaling pathway (18), and they are positively correlated with tumor initiation and adverse prognosis (19). There have been documented interactions between fibroblasts and tumors, enabling them to regulate tumor cell metabolism and modulate associated immune functions, making them conducive to tumor growth (20, 21). Additionally, numerous studies have found that tumor-associated fibroblasts can participate in regulating the stemness and epithelial-mesenchymal transition of tumor cells, thereby promoting tumor progression (22–24). However, there is relatively limited literature available on the interaction between fibroblasts and ccRCCs tumor cells at the single-cell level. This study delves into elucidating the functional roles and underlying mechanisms of Myofibroblasts and EPCs in ccRCCs at the single-cell level. Through meticulous subpopulation stratification of Myofibroblasts and EPCs, we explore intricate cell-cell interactions, specifically focusing on those occurring between the crucial subpopulation C3 and tumor cells within EPCs. Simultaneously, we construct prognostic models linked to these interactions, subsequently subjecting them to rigorous experimental validation. Based on these research findings, we hypothesize a strong correlation between the progression of ccRCCs and the interplay pathways of oxidative phosphorylation and Myofibroblasts, with the pathway gene MPZL1 playing a pivotal role. These studies contribute to a deeper understanding of ccRCCs’ proliferation and recurrence at the single-cell level, aiming to provide insights for the design of better therapeutic targets and prognostic models for ccRCCs treatment.

## Methods

### Data processing and download

We acquired the neoplastic specimens and adjacent tissues from six patients diagnosed with Clear Cell Renal Cell Carcinomas (ccRCCs) through the European Nucleotide Archive (ENA) database (https://www.ebi.ac.uk/ena/browser/home). The Project number is PRJNA705464. Single-cell samples were extracted. Gene expression quantification using RNA-Seq (HTSeqFPKM) and clinical sample data for ccRCCs were retrieved from The Cancer Genome Atlas (TCGA) website (https://portal.gdc.cancer.gov) and subsequently processed and normalized utilizing R software (R 4.3.0).

### Quality control

The R package DoubletFinder was employed for the filtration and removal of low-quality cellular data, including instances of doublet cells and multi-cellularity in the samples (25, 26). The filtering criteria encompassed the following parameters: 1) Total cellular gene transcripts (nCount) ranging from 500 to 80,000; 2) Total cellular gene counts (nFeature) between 200 and 6,000; 3) The proportion of mitochondrial genes less than 25%; 4) The proportion of erythrocyte gene counts below 5%, with a count proportion also below 5%.

### Clustering and annotation

We utilized the NormalizeData function within the R package Seurat to standardize the curated single-cell data (27). Subsequently, we computed the variance and standard deviation for each gene through the FindVariableFeatures function, selecting the top 2000 genes as highly variable based on the genes’ average expression and the extent of their dispersion (28, 29). All genes underwent centering via the ScaleData function, and the cyclical effects of distinct cells were assessed using the CellCycleFeatures function. The RunPCA function facilitated dimensionality reduction based on the expression of the top 2000 high-margin genes, followed by the mitigation of batch effects from the samples using the RunHarmony function from the R package harmony.

For clustering the data after dimensionality reduction, the FindNeighbors and FindClusters functions from the Seurat package were employed. Integration and annotation of distinct cell clusters post-clustering were conducted with reference to the CellMarker database (http://bio-bigdata.hrbmu.edu.cn/CellMarker/) and the SingleR function. Subsequent corrections were applied through a meticulous review of pertinent literature. Sample source analysis for diverse large cell clusters was executed, and the identification and annotation of differentially expressed MARKER genes among these clusters were performed using the FindAllMarkers function (30, 31).

### Analysis of cell sources and enrichment of large populations

We scrutinized the cellular origins and relative sizes of the principal clusters within ccRCCs. A comparative analysis was conducted on the total number of transcribed genes and the transcriptional profile across different major clusters, visually represented through column line graphs and UMAP (32, 33). Furthermore, we identified differential genes (DEGs) among the cells of these major clusters, necessitating DEGs to be detected in a minimum of 25% of the cells, with a false discovery rate (FDR) below 0.05, | logFCfilter | exceeding 1. The DEGs identified in the major clusters underwent Gene Ontology (GO) enrichment analysis.

Additionally, we independently computed DEGs between the two cellular macropopulations of Myofibroblasts and Epithelial Cells (EPCs) and other macropopulations. This analysis was carried out using the Kyoto Encyclopedia of Genes and Genomes (KEGG) database (c2.cp.kegg.v7.5.1.symbols.gmt). The marker genes retrieved from the database underwent filtration and analysis for Gene Set Enrichment Analysis (GSEA), with statistical significance set at FDR < 0.05.

### Analysis of cellular metabolism in large populations

We computed the expression levels of stemness genes across various major clusters within ccRCCs. Additionally, we evaluated cellular metabolic pathways within the major clusters of ccRCCs and distinct cellular sources using the R package scMetabolism. The outcomes were presented in the form of heatmaps, showcasing the top 20 pathways. Further exploration involved the selection of the top three pathways with the highest scores, visualizing their expression across major populations through UMAP. A comparative analysis was performed on the top three metabolic pathways within major populations and diverse sources of ccRCCs, illustrated in the form of violin plots.

### Myofibroblasts subgroup correlation analysis

We employed the FindNeighbors function and FindClusters within the Seurat package to reclustering the downscaled Myofibroblasts cell clusters. Subsequently, we identified marker genes for each subgroup based on the percentage of expression and the frequency of expression, and conducted chromosome copy variation analysis (inferCNV) in different subpopulations of Myofibroblasts. We delved into the tissue origin and cellular staging of cells within each subpopulation, visualizing the findings through UMAP. Furthermore, we conducted a comparative analysis of the tissue origin and cellular staging ratios among different subpopulations. Staging scores, including G2M.Score and S.Score, were computed and compared across subpopulations. Additionally, we explored and compared the nFeature and nCount of genes within each subgroup. Disparities in G2M.Score, S.Score, nFeature, and nCount were calculated and compared across various subpopulations.

### Trajectory and correlation analysis of myofibroblasts subgroups

To investigate the differentiation and evolutionary processes among cells within each subpopulation of Myofibroblasts, we conducted a proposed time-series analysis. Initially, we predicted the relative differentiation status of each cell subpopulation using the R package Cytotrace, assigning scores within the interval of 0-1, where higher scores indicate heightened cell stemness. Subsequently, the developmental time of each cell subpopulation was calculated and organized by the R package monocle, unveiling the temporal transcriptional dynamics of marker genes within each cell subpopulation.

Following chronological ordering, cells were categorized into three periods, and the sequential changes of subpopulation cells, along with cells originating from different sources within the subpopulation, were computed for each period. Ultimately, leveraging slingshot, cell clustering clusters, and spatial dimensionality reduction information, we executed cell differentiation genealogy construction and pseudo-temporal extrapolation. This approach validated the temporal order of different subpopulations at an elevated level and calculated the proportionality between cell staging and diverse subpopulations according to slingshot.

We further computed and compared the stemness genes of cells within each subpopulation, visualizing the results. Through an amalgamation of tissue origin and cell staging information of subpopulation cells, coupled with the analysis of subpopulation cell stemness and associated genes, we identified the C3 subpopulation as a potential key subpopulation. Subsequently, using the pyscenic algorithm, we calculated the top 5 transcription factors for each subpopulation, delving into the overall distribution and regulatory regions of the pivotal C3 subpopulation.

### Subpopulation cell interaction analysis

To elucidate the cell-cell interactions among subpopulations and larger populations, and to delve further into the interaction dynamics between the pivotal C3 subpopulation of Myofibroblasts and tumor cells, we conducted Copy Number Variation (CNV) analysis on EPCs cells, distinguishing tumor cells within EPCs based on the count of copy variants. Subsequently, utilizing the R package cellchat, we analyzed the overall count of cell-cell interactions and their respective strengths. These interactions were further categorized into those involving tumor cells and those with other cell types. Based on the interaction results, we scrutinized the crucial interaction pathway MPZ between C3 and tumor subpopulations.

### Clinical correlation and independent prognosis analysis of C3 subpopulations

The R software was employed to determine the intersection of the identified marker genes within the C3 subgroup with both ccRCC tumor tissues and normal tissues. Samples from ccRCCs with incomplete clinical data were excluded. Subsequently, the intersected genes were merged with the standardized clinical data of ccRCCs. Univariate COX risk regression analysis was conducted using the coxph function within the R package survival. This analysis was validated through the application of the Least Absolute Shrinkage and Selection Operator (LASSO)-penalized Cox regression (34–36). Finally, multivariate COX risk regression analysis was performed to identify prognostic differential genes.

The risk score for each sample was calculated (risk score = Xλ, where X represents the relative expression level of prognostic genes and coefλ is the coefficient) (37, 38). Samples were categorized into high and low-risk groups based on their risk scores. Coefficient values of prognostic genes were computed, and the distribution of these genes in the high and low-risk groups was visualized through a heatmap. Kaplan-Meier curves illustrated the survival at different time points in the high- and low-risk groups (39, 40), while the specificity and sensitivity of prognostic genes were further validated using time-dependent receiver operating characteristic (ROC) curves (41, 42).

Additionally, the correlation between specific prognostic genes and risk scores was calculated and demonstrated. To construct the prognostic model, information on race, gender, age, and tumor stage in the samples was incorporated. A nomogram was created using the R package rms, combining the risk scores to predict the prognosis of ccRCC patients (43). This model was finally validated through ROC curves and decision curve analysis (DCA).

### Immune correlation analysis and enrichment analysis

We computed and authenticated immune infiltration in the high and low-risk groups utilizing the xCell and CIBERSORT deconvolution algorithms to compare and validate immune infiltration, respectively. The correlation of immune cells with the risk score and prognostic genes was explored. TIDE scores were calculated for high and low-risk groups, and the correlation of prognostic genes and risk scores with immune checkpoint genes was investigated. Tumor immune microenvironment-specific scores of the high and low-risk groups were also calculated and compared.

To delve into the correlation mechanism of the high and low-risk groups, we further filtered the differential genes using the R package limma, employing filtering conditions of |log FC| > 1 and a threshold value of FDR (BH) corrected P.adj <0.05. GO and KEGG enrichment analyses were conducted using the R package clusterProfiler, encompassing biological processes, cellular components, and molecular functions in GO enrichment analyses (44, 45).

### Cell culture

The 786-O and CAKI-1 cell lines were procured from the American Type Culture Collection (ATCC). Both cell lines underwent cultivation in F12K medium supplemented with 10% fetal bovine serum (Gibco BRL, USA) and 1% streptomycin/penicillin. PRMI1640 medium (Gibco BRL, USA) was utilized for the CAKI-1 cell line. Standard conditions of incubation were maintained at 37°C, 5% CO2, and 95% humidity.

### Cell transfection

MPZL1 knockdown was achieved through the utilization of small interfering RNA (siRNA) constructs obtained from GenePharma, Suzhou, China. The transfection protocol closely adhered to the steps outlined for Lipofectamine 3000RNAiMAX (Invitrogen, USA). Cells were seeded in 6-well plates at 50% confluence and subsequently transfected with negative control (si-NC) and knockdown (Si-MPZL1-1 and Si-MPZL1-2). Each transfection process was carried out utilizing Lipofectamine 3000RNAiMAX (Invitrogen, USA).

### Cell viability assay

Cell viability of 786-O and CAKI-1 cells post-transfection was assessed using the CCK-8 assay. Cell suspensions were seeded at a density of 5*103 cells per well in 96-well plates and cultured for 24 hours. Subsequently, 10 μL of CCK-8 reagent (A311-01, Vazyme) was added to each well and incubated in the dark at 37°C for 2 hours. Cell viability was determined by measuring absorbance at 450 nm using an enzyme marker (A33978, Thermo) on days 1, 2, 3, and 4. The mean OD values were computed and presented on a line graph.

### 5-Ethyl-2 ' -deoxyuridine proliferation assay

Transfected 786-O and CAKI-1 cells were seeded in 6-well plates at a density of 5 × 103 cells per well and allowed to incubate overnight. A 2x EdU working solution was prepared by incorporating a 10 mM EdU solution in serum-free medium, which was subsequently added to the cell culture and incubated at 37°C for 2 hours. Following incubation, the medium was aspirated, and cells were washed with PBS before fixation with 4% paraformaldehyde for 30 minutes. Subsequent treatment involved exposure to glycine (2 mg/ml) and 0.5% Triton X-100 for 15 minutes. Cells were then incubated with 1 ml 1X Apollo and 1 ml 1X Hoechst 33342 for 30 minutes at room temperature. Cell proliferation was quantified through fluorescence microscopy.

### Wound healing

The transfected cells were seeded in 6-well plates and cultured until reaching a cell density of 95%. A sterile 200 μL pipette tip was initially employed to create a straight-line scratch across the cell layer in the culture wells, followed by gentle rinsing with PBS. Subsequently, the medium was replaced to facilitate ongoing cell culture. Photographs of the scratch were captured at the identical location at 0 hours and 48 hours, and the width of the scratch was measured.

### Transwell experiment

Cells underwent serum deprivation in medium lacking serum for a duration of 24 hours preceding the experiment. Following treatment involving the addition of matrix gel (BD Biosciences, USA), the cell suspension was introduced into the upper chamber, which contained Costar, while serum medium was introduced into the lower chamber. Subsequent to a 48-hour incubation period in the incubator, cells were fixed using 4% paraformaldehyde and stained with crystal violet to assess their invasive capability.

## Results

### ccRCCs large population cell categorization

**Figure 1:**
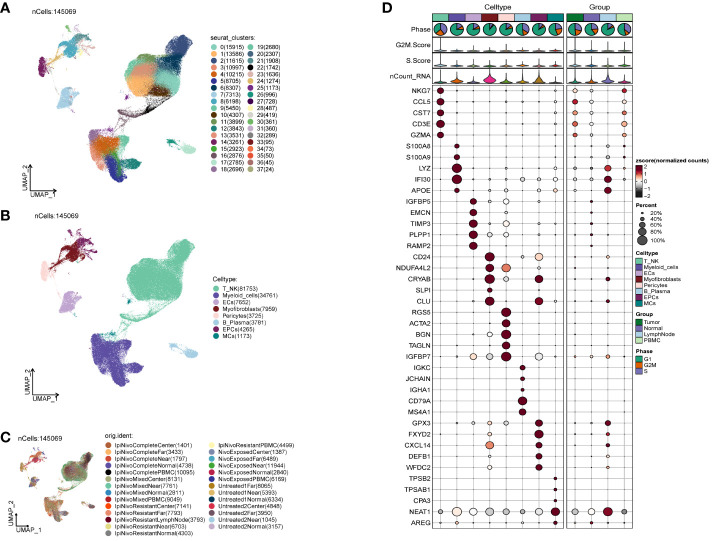
Classification of ccRCCs cells into large clusters. **(A)** 6 patients with ccRCCs ccRCCs tumor and surrounding tissues were classified into 38 clusters after single cell clustering; **(B)** According to different MARKER genes, the ovaries were annotated into T_NK, Myeloid_cells, ECs, Myofibroblasts, Pericytes, B_Plasma, EPCs, MCs respectively, a total of 8 **(C)** Distribution of each sample source. **(D)** Each population of cells exhibits the top five marker genes in ccRCCs.

We acquired the single-cell data of ccRCCs from the ENA database (PRJNA705464), encompassing Tumor, Normal, LymphNode, and Peripheral Blood (PBMC) samples. Utilizing the R package DoubletFinder, we eliminated duplicated cells and subsequently filtered out subpar cells in the samples (**Supplementary Figure 1A**). Cell staging tests revealed a concentrated distribution in the PCA plot, indicating minimal impact on the study results (**Supplementary Figure 1B**). Selecting the top 2000 highly variable genes based on expression and dispersion (**Supplementary Figure 1C**), we downscaled them using RunPCA and retained the top 30 dimensions for subsequent analysis (**Supplementary Figures 1D, E**). Exploration of the top 10 highly variable genes for each of the first 9 dimensions was visualized (**Supplementary Figure 1F**). We categorized ccRCCs into 37 clusters (**Figure 1A**) and annotated them using the R package singleR and CellMarker databases. After further correction based on relevant literature, we categorized them into 8 major cell clusters (**Figure 1B**), namely T_NK(81753), Myeloid_cells(34761), ECs(7652), Myofibroblasts(7959), Pericytes(3725), and B_Plasma(3781).

During the study, Myofibroblasts, Epithelial Cells (EPCs)(4265), and MCs(1173) emerged as crucial cell groups in the tumor. Reviewing literature on the annotated cell populations, we identified EPCs as pivotal cells in the tumor, with a close association with Myofibroblasts. Disparities in sample distribution among different cells were observed, possibly linked to the individual source of the samples and the location from which tumor tissues were selected (**Figure 1C**). Examination of the top 5 MARKER genes in each subpopulation (**Figure 1D**) revealed that MARKER genes of EPCs also exhibited some expression in LymphNode cells.

### Analysis of cell origin and gene transcription correlation in large populations

**Figure 2:**
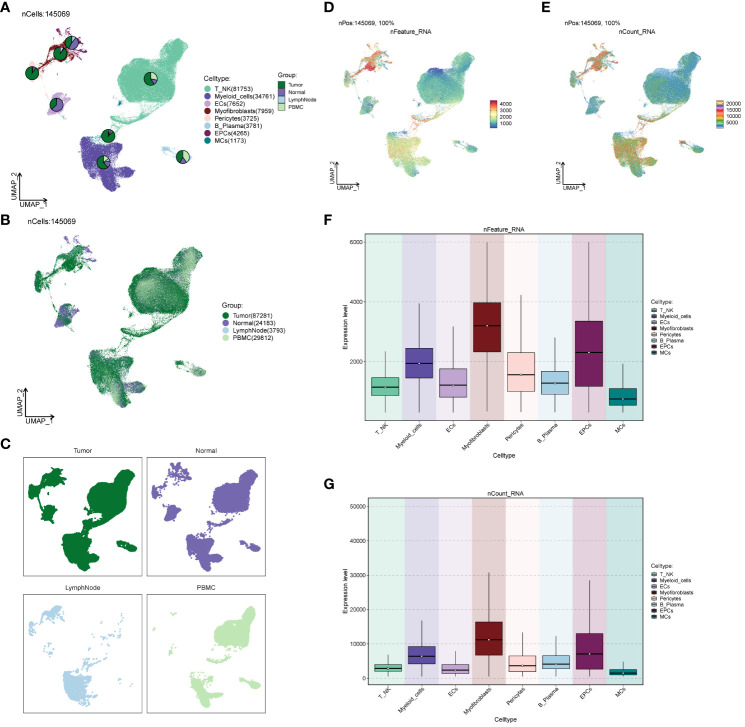
Source site and transcriptional analysis of the major clusters of cells. **(A–C)** ccRCCs major clusters of cells source site and distribution plot; **(D, E)** ccRCCs major clusters of cells nFeature and nCount UNMP distribution plot; **(F, G)** ccRCCs major clusters of cells nFeature and nCount expression level box line plot.

We investigated the spatial distribution of the cell population in ccRCCs (**Figures 2A–C**) and noted a relatively high proportion of tumor cells within Myofibroblasts. In contrast, EPCs displayed a mixed composition, including a significant presence of normal tissue cells. Acknowledging the necessity to extract tumor cells from EPCs in subsequent analyses, we proceeded accordingly. Examination of nCount and nFeature (**Figures 2D–G**) revealed higher total transcript numbers and gene quantities in EPCs and Myofibroblasts cells, indicating elevated cellular activities and functions in these two locations.

### Enrichment function analysis

**Figure 3:**
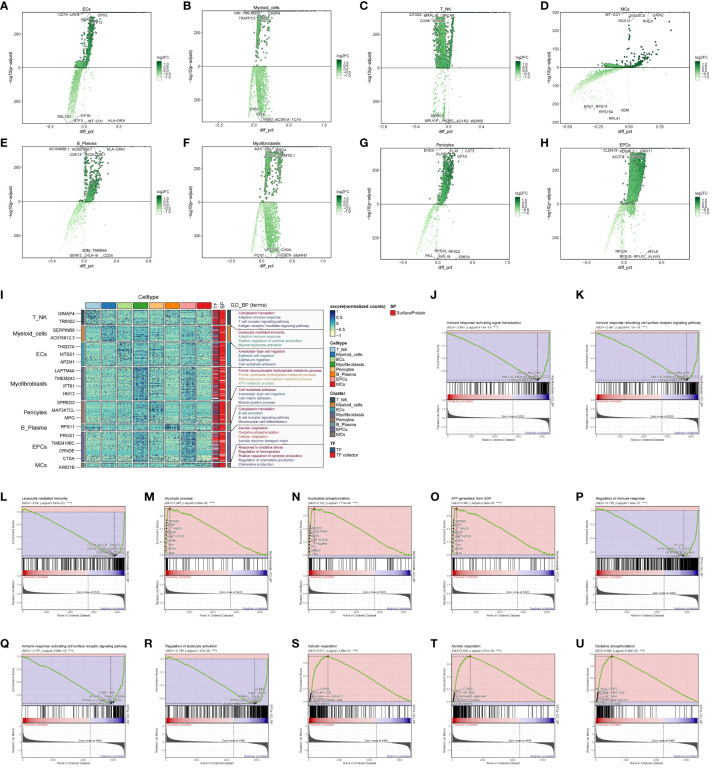
Enrichment analysis of major clusters of cells. **(A–H)** Differential gene distribution plot of ccRCCs major population cells versus other cells, in which the top 5 selected genes from each of the high and low expression genes are shown; **(I)** GO enrichment analysis plot of ccRCCs major population cells; **(J–O)** GSEA enrichment analysis plot of Myofibroblasts, with 5 pathways selected from each of the low expression group and the high expression group. **(P–U)** GSEA of EPCs Enrichment analysis plot with 5 pathways selected from each of the low expression group vs. high expression group.

To explore the functional attributes of the cell populations in the large cohort of ccRCCs, we scrutinized the differential genes between these cells and others (**Figures 3A–H**). Subsequently, we conducted GO enrichment function analysis based on the identified differential genes (**Figure 3I**). Notably, both EPCs and Myofibroblasts exhibited enrichment in oxidative respiration, with pathways such as Aerobic respiration and Oxidative phosphorylation significantly enriched in EPCs, while ATP metabolic process and Purine ribonucleoside triphosphate metabolic process were notably enriched in Myofibroblasts.

To delve deeper, we performed GSEA enrichment analysis for EPCs and Myofibroblasts. In Myofibroblasts (**Figures 3J–O**), the low-expression group showed significant enrichment in pathways related to immune response-activating signal transduction, immune response-activating cell surface receptor signaling pathway, and leukocyte-mediated immunity. Conversely, the high-expression group exhibited enrichment in pathways like Glycolytic process, Nucleotide phosphorylation, and ATP generation from ADP. In EPCs (**Figures 3P–U**), the low expression group displayed enrichment in pathways related to the regulation of immune response, immune response-activating cell surface receptor signaling pathway, and regulation of leukocyte activation. In contrast, the high expression group showed enrichment in pathways associated with cellular respiration, aerobic respiration, and oxidative phosphorylation.

These findings suggest that Myofibroblasts and EPCs share similar GSEA-enriched pathways, hinting at a potential connection between the high- and low-expression groups. We posit that the oxidative response of Myofibroblasts may drive the suppression of immune function.

### Cell stemness and metabolic analysis

**Figure 4:**
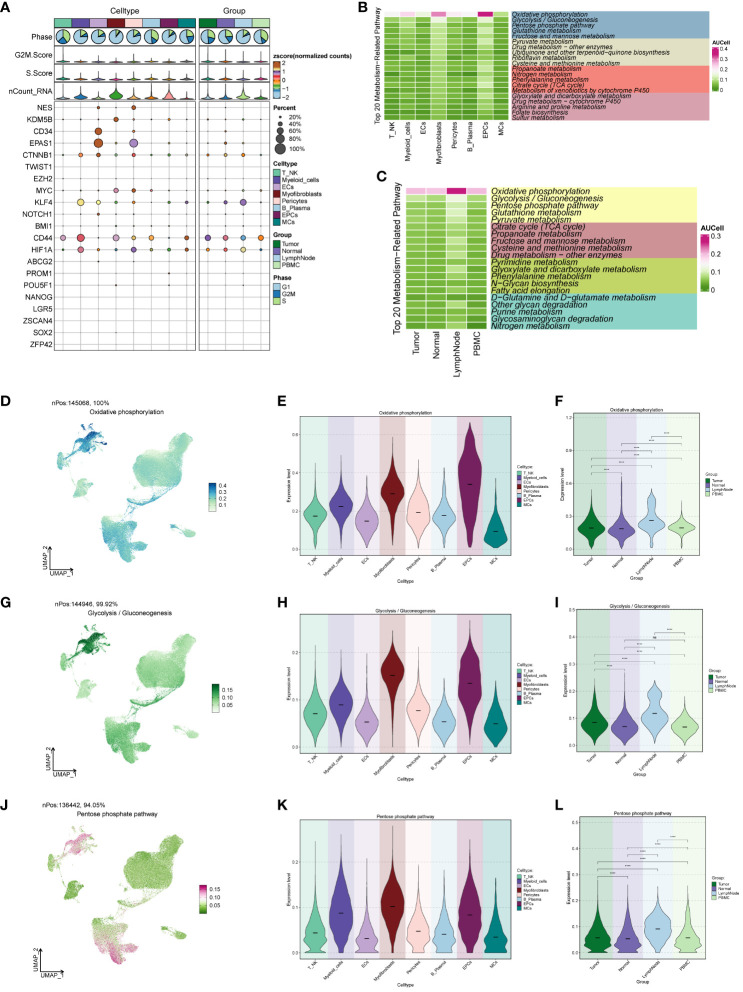
ccRCCs large population of cell stemness genes and metabolic pathways map. **(A)**Analysis of stemness genes in large populations of ccRCCs; **(B)** Top 20 metabolic pathways in large populations of ccRCCs; **(C)** Top 20 metabolic pathways in cells of different positional origins of Tumor, Normal, LymphNode, and PBMC; **(D–L)** Distribution of UNMPs for the three pathways “Oxidative phosphorylation, Glycolysis/Gluconeogenesis, Pentose phosphate pathway” and violin maps of the scores of the three pathways in the large population of ccRCCs with different locations of origin. **** means p <0.0001; ns means no statistical difference.

We conducted an analysis of cell stemness genes in the large cell populations, identifying a high score for CD44 in Myofibroblasts (**Figure 4A**). Further exploration of metabolic function differences between large cell populations and cells of different origins (**Figures 4B, C**) revealed significantly higher scores for cellular metabolic pathways, including Oxidative phosphorylation, Glycolysis/Gluconeogenesis, and the Pentose phosphate pathway—all associated with oxidative respiration. To provide a comprehensive view, we compared the distribution and scores of these three pathways in each cell population (**Figures 4D–L**), observing their pronounced significance in both EPCs and Myofibroblasts. This aligns with our earlier findings from GO and GSEA enrichment analyses.

### Subclustering of myofibroblasts

**Figure 5:**
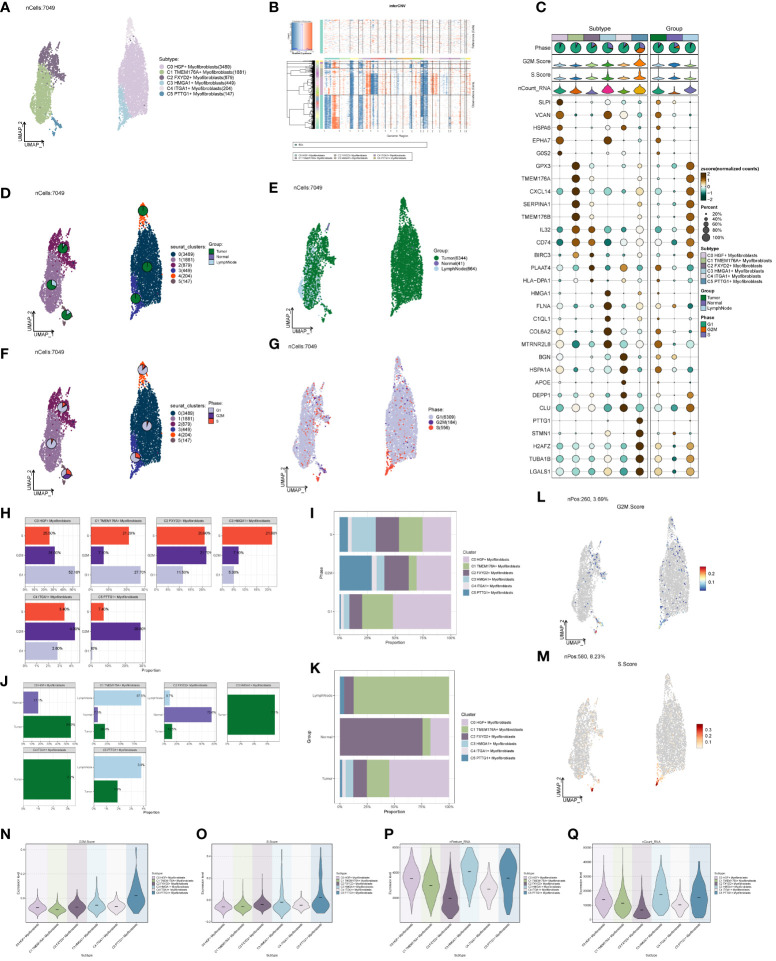
Subpopulation of ccRCCs cells and correlation analysis. **(A)** UNMP distribution of subpopulation cells; **(B)** Heatmap of CNV situation of subpopulation cells; **(C)** Expression of the first 5 marker genes in each subpopulation cells; **(D, E)** UMAP map of the distribution of source cells in different locations of Tumor, Normal, LymphNode; **(F, G)** UNMP map of the distribution of subpopulation of cells in each staging cells of G2M, S, G1; **(H, I)** G2M, S, G1 Proportional distribution between cells and subpopulations of cells in each stage; **(J, K)** Proportional distribution between subpopulations of cells and cells originating from different locations of Tumor, Normal, and LymphNode; **(L)** UNMP distribution of G2M.Score; **(M)** S.Score UNMP distribution; **(N–Q)** G2M.Score, S.core, nFeature and nCount Violin graph representing score.

Distinct Myofibroblast subpopulations were identified as C0, C2 (FXYD2+), C3 (HMGA1+), C4 (ITGA1+), and C5 (PTTG1+) (**Figure 5A**). We calculated copy number variations (CNVs) of these subpopulations and the respective marker genes (**Figures 5B, C**). The cellular origin investigation revealed higher tumor tissue content in C0, C3, and C4 (**Figures 5D, E**). Assessing the proportionality of subpopulations with respect to cell origin (**Figures 5J, K**) indicated the highest amount and purity in C3 tumor tissues. Examining cell stage distribution (**Figures 5F, G**) showed a relatively high proportion of G2M and S stages in C3 and C5. The cell stage ratio graph (**Figures 5H, I**) highlighted significantly higher S stage in C3 compared to G2M and G1 stages, indicating vigorous cellular DNA synthesis, replication, and proliferative ability. G2M.Score and S.Score distributions (**Figures 5L–O**) revealed higher scores for both C3 and C5 subpopulations. Additionally, comparing nFeature and nCount values of different subpopulation cells (**Figures 5P, Q**) demonstrated significantly higher values in C3 compared to other subpopulations.

### Proposed time series analysis of subpopulation cells

**Figure 6:**
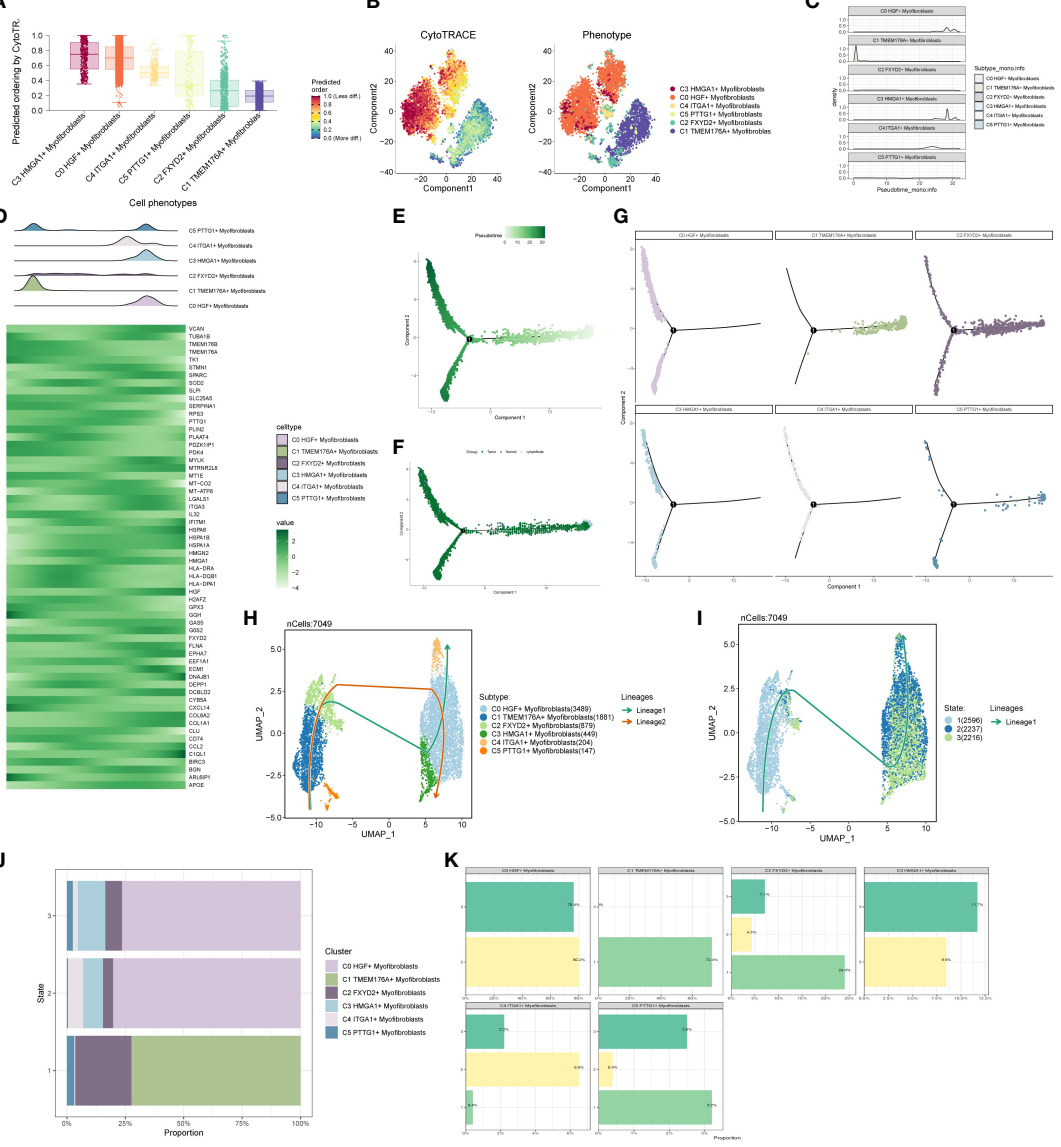
Proposed time-series analysis of subpopulation cells. **(A, B)** Cytotrace scores of subpopulation cells; **(C, D)** monocle-based time trajectory maps of each subpopulation and marker genes; **(E, F)** monocle-based time trajectory staging, which can be categorized into three periods; **(G)** distribution of subpopulation cells with time trajectories based on monocle; **(H, I)** slingshot-based time staging maps of subpopulation cells, which can be divided into three periods with two trajectories of merit; **(J, K)** distribution of the ratio of subpopulation cells to time staging period.

We predicted the differentiation of individual subpopulations by the R package Cytotrace and found that the C3 group had the highest degree of differentiation, followed by C0 and C4 (**Figures 6A, B**). To understand the temporal trajectory relationship between cell subpopulations, we calculated the relative temporal order of the individual subpopulations by the R package monocle (**Figure 6C**), and found that the C1 subpopulation was at the starting point, whereas the C3 and C0 subpopulations were near the end point. We also analyzed the temporal trajectory relationship of marker genes of each subpopulation (**Figure 6D**) and explored the temporal trajectories of different cellular origins of each subpopulation and individual subpopulations (**Figures 6E–G**), and we found that subpopulations C1 and C2 were more at the beginning of the trajectory, while C0 and C3 were mostly located at the end of the trajectory. We also validated the intercellular trajectories by slingshot and found that they could be divided into three periods and there were two time trajectories (**Figures 6H, I**), while C3 was at the middle and end of trajectory 1 and at the tail end of trajectory 2. We also calculated the proportional relationship between different subpopulations and different periods with each other (**Figures 6J, K**), and found that the C3 subpopulation was in stages 2 and 3, and mainly existed in stage 3, indicating that the C3 subpopulation was at the end of the time trajectory compared with the C0 subpopulation, which mostly represented that the tumors had already completed the transition from normal cells to malignant cells, and might have a high degree of invasion and value-added ability, as well as drug resistance. With the above analysis, we consider the C3 subgroup as the key subgroup.

### Analysis of cell stemness genes and transcription factors in subpopulations

**Figure 7:**
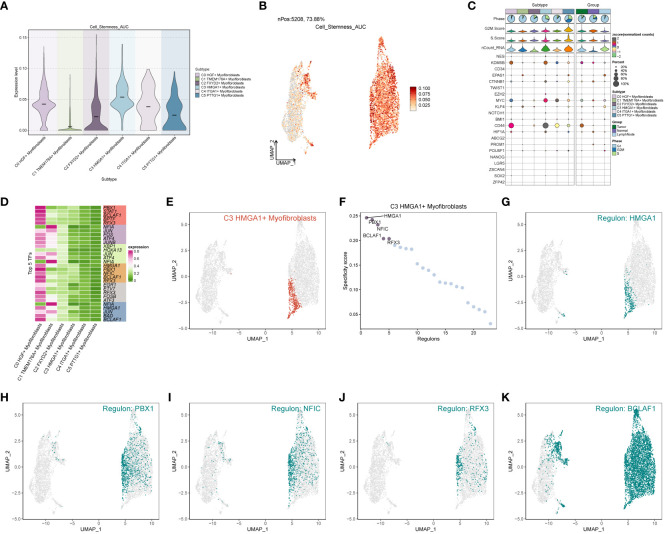
Subpopulation cell stemness gene and transcription factor analysis. **(A)** Cellular stemness gene expression level violin plot for each subpopulation of cells in Myofibroblasts; **(B)** UMAP distribution of stemness gene expression level in subpopulation of cells; **(C)** Stemness gene expression in each subpopulation of cells in Myofibroblasts; **(D)** Top 5 transcription factors of each subpopulation of cells in Myofibroblasts; **(E–K)** Top 5 transcription factors UMAP distribution graph.

In the analysis of cell stemness genes (**Figures 7A–C**), it was discerned that the C3 subpopulation exhibited the most robust scores for cell stemness genes, with CD44 showing heightened expression in both the C3 subpopulation and tumors. Furthermore, an examination of transcription factors in each subpopulation revealed that the top 5 transcription factors in the C3 subpopulation were HMGA1, PBX1, NFIC, BCLAF1, and RFX3 (**Figure 7D**). A detailed exploration of the expression of these 5 transcription factors across cellular subpopulations (**Figures 7E–K**) highlighted the prominent expression of the transcription factor HMGA1, particularly in the predominant C3 subpopulation.

### Analysis of cellular interactions

**Figure 8:**
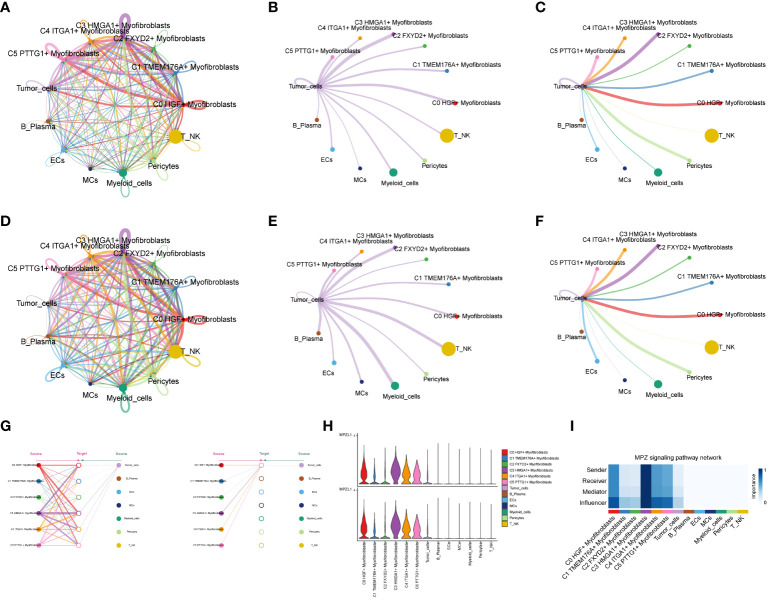
Interaction analysis between subpopulation cells and large population cells. **(A-C)** Number of interactions between Myofibroblasts subpopulation cells and ccRCCs large population cells graph, where A is the number of interactions between all cells, B figure tumor cells as source, C figure tumor cells as target, the thicker line represents the more number; **(D–F)** Interaction between Myofibroblasts subpopulation cells and ccRCCs large population cells Intensity map, where D is the number of interactions among all cells, **(E)** means that Tumor cells are the source, and **(F)** means that Tumor cells are the target; thicker lines represent higher intensity; **(G)** MPZ signaling pathway network pathway interactions between subpopulations and large populations of cells; **(H)** MPZL1 gene expression in subpopulations and large populations; **(I)** Scores of action sites in MPZ signaling pathway network pathway in each subpopulation and large populations.

Through the computation of CNV values in EPCs, we pinpointed the tumor cell populations within EPCs (**Supplementary Figure 2**). Subsequent exploration into the number of cellular interactions within subpopulations of cells and the broader cell populations (**Figures 8A–C**) unveiled that tumor cells exhibited the highest degree of contact with the C3 subpopulation. Additionally, in the assessment of interaction strength (**Figures 8D–F**), tumor cells demonstrated more robust connections with the C3 subpopulation compared to other cells. Our deduction is that the C3, identified as a key subpopulation of Myofibroblasts, likely harbors the most potent interactions with tumor cells. Further scrutiny into the interactions involving the C3 subpopulation and other subpopulations (**Figures 8G–I**) brought to light that the MPZ signaling pathway network held particular significance among the subpopulations. Notably, MPZL1, a pivotal gene in this pathway, scored the highest within the C3 subpopulation.

### Cellular prognostic analysis

**Figure 9:**
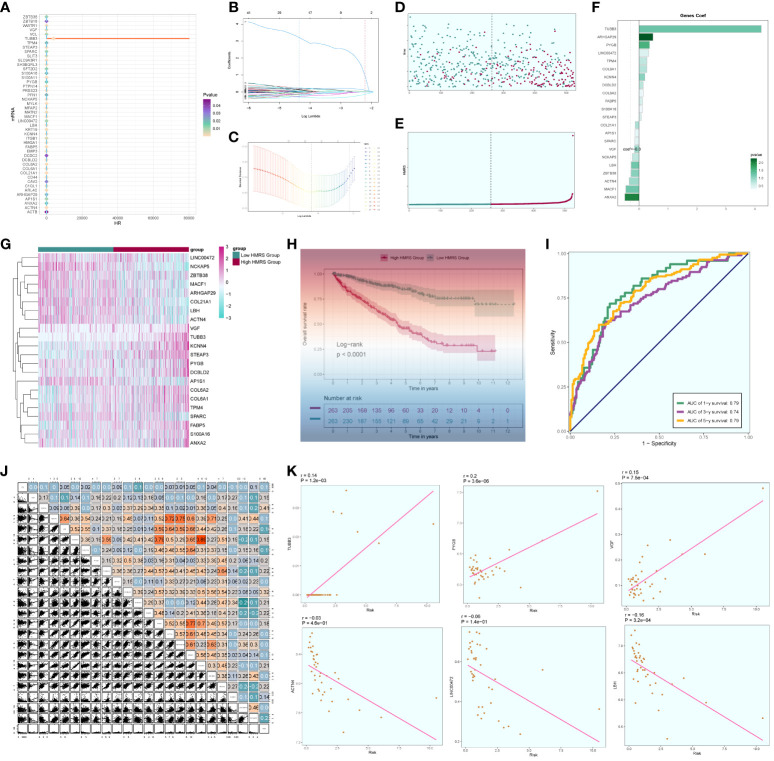
Independent prognostic analysis. **(A)** 46 prognosis-related differential genes; **(B)** Distribution of LASSO analysis coefficient spectra of 22 prognosis genes; **(C)** Optimal cross-validation of parameter selection in LASSO regression; **(D)** Survival time and survival status plots of patients in different risk groups over time; **(E)** Classification of patients into high- and low-risk groups based on risk scores; **(F)** Coef values of prognosis-related genes; **(G)** Heat map of prognosis-related gene distribution; **(H)** Kaplan-Meier prognostic analysis curves for high and low risk groups; **(I)** Time-dependent ROC curves with area under the curve (AUC) of 0.79, 0.74, 0.79 at 1, 3, and 5 years; **(J)**. Correlation analyses between genes and risk scores and OS; **(K)** Dot plots of the top 6 prognostic genes that had a strong correlation with the risk score.

**Figure 10:**
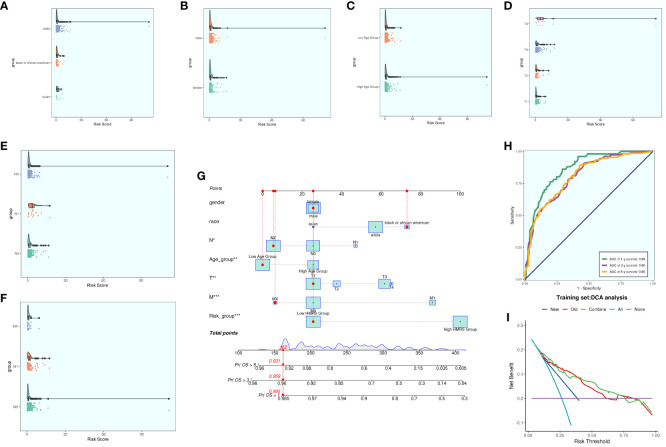
Clinical correlation analysis. **(A–F)** analysis of the correlation of risk scores with racial factors, gender, age, and tumor stage T, N and M **(G)** Nomogram plots of patients with ccRCCs at 1, 3, and 5 years; **(H)** time-dependent ROC plots, with AUCs of 0.86, 0.80, and 0.80 at 1, 3, and 5 years, respectively; **(I)** DCA analysis of prognostic models Figure.

We conducted a comprehensive analysis of ccRCCs by intersecting differentially expressed genes from the TCGA database with C3 subpopulation marker genes. Through univariate COX risk regression analysis, we identified 46 prognostically relevant differential genes (**Figure 9A**). Validation with LASSO COX risk regression analysis confirmed the stability and reliability of these genes (**Figures 9B, C**). Subsequently, multivariate COX risk regression analysis yielded 22 final prognostic genes, including high-risk genes such as S100A16, FABP5, COL6A2, DCBLD2, KCNN4, COL6A1, TPM4, LINC00472, PYGB, ARHGAP29, TUBB3, and STEAP3, and low-risk genes such as ANXA2, MACF1, ACTN4, ZBTB38, LBH, NCKAP5, VGF, SPARC, AP1S1, and COL21A1. Patients were stratified into high- and low-risk groups based on risk scores (**Figures 9D, E**). The survival analysis revealed a progressive increase in the number of patient deaths over time, with the high-risk group exhibiting lower survival rates (**Figures 9D, E**). Examination of coefficient values (**Figure 9F**) showed TUBB3 with the highest score and ANXA2 with the lowest. The expression patterns of prognostic genes in high- and low-risk groups were explored (**Figure 9G**). Kaplan-Meier curve analysis demonstrated significantly lower survival rates in the high-risk group (**Figure 9H**), with a meaningful result (P < 0.0001). ROC curve analysis indicated stable and good predictive performance at 1, 3, and 5 years (**Figure 9I**). We further examined the correlation of different genes with risk score and prognosis (**Figure 9J**) and visualized six genes significantly associated with risk score (**Figure 9K**). Exploration of the correlation of clinical factors with the risk score and construction of a nomogram (**Figures 10A–G**) allowed for predicting the survival rate of ccRCC patients at 1, 3, and 5 years. Validation through ROC and DCA curves demonstrated the model’s high stability, sensitivity, and clinical utility (**Figures 10H, I**).

### Immune correlation analysis

**Figure 11:**
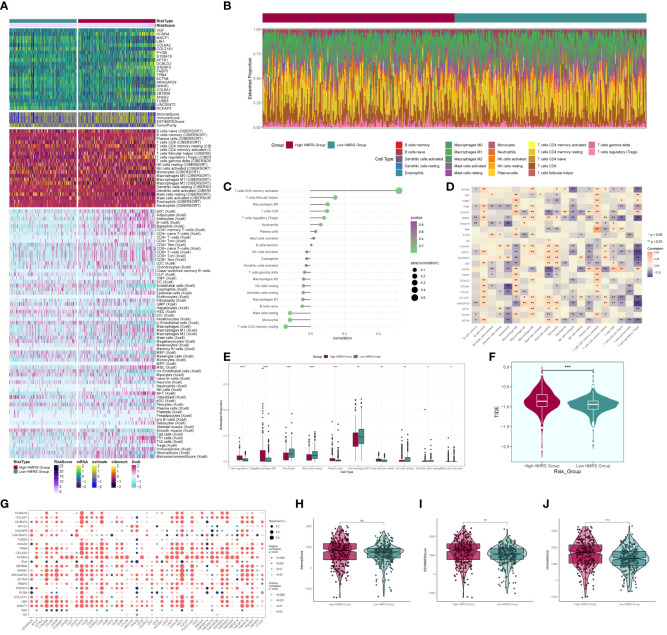
Immune correlation analysis. **(A)** Heatmap of prognostic genes, tumor microenvironment and immune cell profile in high and low risk groups; **(B)** Difference in the proportion of immune cells in high and low risk groups; **(C)** Immune cell risk score relationship score; **(D)** Immune cell correlation with prognostic genes, risk score and OS; **(E)** Difference in immune cell expression between high and low risk groups; **(F)** TIDE score violin plot between high and low risk groups; **(G)** Situation point plot of the relationship between immune checkpoints and prognostic genes, risk scores; **(H–J)** Difference in immune scores, stromal scores, and total scores of tumor microenvironment between high and low risk groups. * means p <0.05; ** means p <0.01; *** means p <0.001; **** means p <0.0001; ns means no statistical difference.

We computed and compared immune cell infiltration in the high- and low-risk cohorts (**Figures 11A, B**) and examined the correlation of immune cells with risk scores and prognostic genes (**Figures 11C, D**). T cells CD4 memory activated showed a positive correlation with RISK, whereas T cells CD4 memory resting exhibited a negative correlation with RISK. Immune cell disparities between high and low-risk groups were also assessed (**Figure 11E**), revealing relatively high scores of Macrophages M2, particularly notable in the low-risk group. TIDE score analysis (**Figure 11F**) indicated a high score in the high-risk group, suggesting a potential for immune escape. Additionally, immune checkpoint score calculation (**Figure 11G**) revealed a predominantly negative correlation with RISK score. Comparing tumor microenvironment-related scores in the high- and low-risk groups (**Figures 11H–J**) showed higher scores in both high-risk cohorts.

### Experimental result

**Figure 12:**
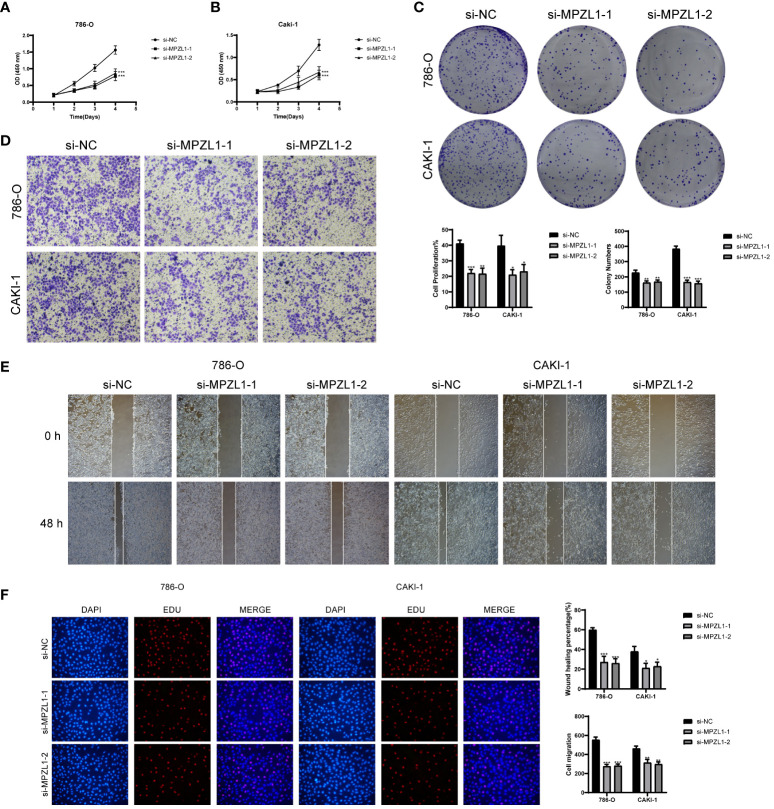
*In vitro* experimental validation of MPZL1. **(A, B)** CCK-8 assay showed that cell viability was significantly reduced after MPZL1 knockdown; **(C)** Colony formation assay showed that the number of colonies in MPZL1 knockdown cells was significantly lower than that in si-NC group; **(D)** Transwell assay showed that MPZL1 knockdown significantly slowed down the growth of 786 -O cells and CAKI-1 cells invasion; **(E)** Scratch assay showed that MPZL1 knockdown significantly slowed down the migration of 786-O cells and CAKI-1 cells; **(F)** EdU staining results showed that MPZL1 knockdown inhibited the proliferation of 786-O cells and CAKI-1 cells. * means p <0.05; ** means p <0.01; *** means p <0.001.

We selected two distinct types of ccRCC cells for the experiment, conducting a comparative analysis between the control group and the knockdown infection group. Remarkably, both cell types exhibited significantly diminished cell viability levels upon MPZL1 knockdown (**Figures 12A, B**). In the cluster formation assay, the number of colonies showed a substantial reduction in both cell types following MPZL1 knockdown (**Figure 12C**). The Transwell assay results (**Figure 12D**) revealed markedly reduced staining regions in cells with MPZL1 knockdown, indicating a diminished invasive capability. In cell scratch assay experiments (**Figure 12E**), the 48-hour scratch width in both cell lines subjected to MPZL1 knockdown was significantly wider compared to the negative control group. The cell proliferation assay (**Figure 12F**) further demonstrated a lower cell count in both cell lines with MPZL1 knockdown compared to the negative control group. Thus, our experimental findings affirm that MPZL1 plays a promotive role in the proliferation, migration, and invasion of ccRCCs, and knockdown of MPZL1 effectively inhibits these processes, restraining the recurrence of ccRCCs.

## Discussion

Renal cell carcinoma stands as the seventh most frequently diagnosed malignant tumor, exhibiting a gradual rise in incidence within developed countries in recent years (46). Among its common clinical subtypes, clear cell renal cell carcinomas (ccRCCs) are associated with diverse clinical prognostic outcomes, revealing metastasis in up to 30% of patients at the onset (47). Surgical intervention, though a common treatment approach, proves ineffective, and there remains a notable risk of recurrence post-surgery (48). Given the limited sensitivity of ccRCCs to radiotherapy drugs (9), the identification of mechanisms and therapeutic targets related to ccRCC progression becomes crucial.

Conducting enrichment analysis and metabolic pathway examination of Myofibroblasts and Endothelial Progenitor Cells (EPCs), we observed elevated scores in oxidation-related pathways and diminished immune function. Notably, the oxidative phosphorylation metabolic pathway exhibited the highest score in both EPCs and Myofibroblasts. A literature review revealed that redox homeostasis underpins normal cellular physiological activities and survival (49). It has been recognized that tumor cells display heightened oxidative processes compared to normal cells (50). Oxidative phosphorylation, a process associated with the generation of reactive oxygen species (ROS), poses a threat to cellular DNA and heightens cancer risk (51–54). Studies by Kuo (55) et al. have indicated that ROS activation correlates with increased blood vessel proliferation, suppression of immune microenvironmental functions, and intensified production of macrophage IFN and IL-6, ultimately hampering the immune response in the tumor microenvironment (56–58). Albiñana (59) et al. identified that reducing oxidative stress and ROS levels through targeting ADRB2 proves beneficial in controlling ccRCC progression. Additionally, Costa (60) et al. established that ROS can induce inflammation, fostering the transformation of fibroblasts into Myofibroblasts, exhibiting characteristics akin to invasive adenocarcinoma-associated fibroblasts.

Thus, we posit that the oxidative phosphorylation pathway and its affiliated pathways play a pivotal role in the progression of clear cell renal cell carcinomas (ccRCCs), impeding the immune microenvironment of these tumors and facilitating their transformation into Myofibroblasts. Consequently, this phenomenon propels the progression and augmentation of the tumor.

Through an exploration of cellular interactions, we discovered that the MPZ signaling pathway network exhibits substantial cellular interactions in crucial subpopulations of Myofibroblasts and tumor cells, with the gene MPZL1 identified as a key player. A comprehensive review of existing literature elucidates that MPZL1 (Myelin protein zero like 1) serves as a binding protein and substrate of tyrosine phosphatase SHP-2 (61), demonstrating widespread expression across various cell types and involvement in essential biological processes and molecular functions (62, 63). Furthermore, the biological impact of MPZL1 in diverse tumors, including ovarian, colorectal, and bladder cancers, has been investigated and corroborated (64–66), highlighting its promotional influence on tumor progression. For instance, Jia et al. (67) observed that MPZL1 promotes the migration of hepatocellular carcinoma cells by inducing the phosphorylation and activation of pre-metastatic proteins. Similarly, Chen (64) reported that elevated expression of MPZL1 stimulates the phosphorylation of Src kinase, facilitating the proliferation and migration of ovarian cancer cells. Additionally, Wang et al. (68) found that overexpression of MPZL1 correlates with the suppression of immune function in lung cancer studies. We also postulate that MPZL1 is intricately linked to the proliferation and migration of ccRCCs, exerting inhibitory effects on the immune microenvironment of tumors, thereby contributing to tumor progression or recurrence. Our experimental results substantiate this hypothesis.

TUBB3 (tubulin beta 3 class III), belonging to the β-tubulin protein family, intricately engages in neurogenesis and microtubule assembly within neuronal cells. Its dynamic interplay involves elongation or depolymerization throughout the cell cycle, exerting substantial influence on cellular morphology, division, and cytoskeletal architecture (69–71). A plethora of studies has consistently emphasized the notable correlation between elevated TUBB3 expression and unfavorable prognoses across various malignancies, including lung, ovarian, and gastric cancers, establishing it as a prognostic indicator for cancer recurrence (72–74). Moreover, certain studies have indicated that high expression of TUBB3 leads to increased drug resistance in tumor cells, which adversely affects patient prognosis (75, 76). Additionally, adenocarcinoma patients with negative TUBB3 expression tend to have longer survival times compared to those with positive expression (77). Furthermore, it has been observed that the TUBB3-associated protein network is involved in certain oxidative stress processes, which may enhance the vitality and drug resistance of tumor cells (78). Experimental research on anti-microtubule chemotherapy drugs has revealed that tumor cells with high TUBB3 expression exhibit stronger resistance to drugs like paclitaxel compared to those with lower expression (79, 80). Our research similarly hypothesizes a close relationship between TUBB3 and oxidative stress-related pathways, potentially promoting the proliferation and recurrence of ccRCCs through this process. This interaction may lead to ccRCCs developing resistance to relevant chemotherapy drugs, ultimately facilitating the progression of ccRCCs.

## Conclusion

In this comprehensive study, a thorough examination of single-cell data related to clear cell renal cell carcinomas (ccRCCs) systematically elucidates the pivotal role of oxidative-related pathways within the diverse cellular milieu of ccRCCs. Simultaneously, we meticulously investigate the critical involvement of these pathways in myofibroblasts and endothelial progenitor cells (EPCs). Additionally, a detailed exploration is conducted into the interactive pathways between key subgroups, such as the C3 HMGA1+ Myofibroblasts in myofibroblasts, and tumor cells within EPCs. This comprehensive analysis extends to the investigation of the essential gene MPZL1, culminating in the construction of a ccRCCs prognostic model intricately linked to these pathways. We posit that both MPZL1 and the oxidative stress pathway harbor the potential to emerge as critical therapeutic targets for the treatment and resistance against recurrence in ccRCCs. This assertion is substantiated through empirical validation, specifically demonstrating the concrete impact of MPZL1 on ccRCCs. Our research has confirmed the significant roles played by Myofibroblasts and the key gene MPZL1 in the progression of ccRCCs, providing novel insights for future studies on ccRCCs. We have discovered that targeting MPZL1 and the oxidative phosphorylation pathway could serve as potential key targets for the treatment of ccRCCs progression and recurrence. This finding opens up new directions for the treatment and prognosis diagnosis of ccRCCs in the future. In conclusion, our discoveries explore the mechanisms underlying the proliferation and recurrence of ccRCCs and shed light on prognostic markers and therapeutic targets for ccRCCs.

## Data availability statement

The original contributions presented in the study are included in the article/**Supplementary Material**. Further inquiries can be directed to the corresponding author.

## Author contributions

WZ: Conceptualization, Data curation, Formal analysis, Investigation, Methodology, Project administration, Resources, Software, Validation, Visualization, Writing – original draft, Writing – review & editing. ZL: Conceptualization, Data curation, Formal analysis, Investigation, Methodology, Project administration, Resources, Software, Validation, Visualization, Writing – original draft, Writing – review & editing. WT: Funding acquisition, Supervision, Writing – review & editing.

## Supplementary material

Supplementary Figure 1 Quality control and dimensionality reduction clustering of data. **(A)** Quality-controlled and filtered single-cell data of ccRCCs. **(B)** Filtered ccRCCs cell stage examination showing G1, G2M, and S stages. **(C)** Selection of the top 2000 highly variable genes based on gene expression and dispersion, with the top 10 highly variable genes indicated. **(D)** RunPCA dimensionality reduction plot showing the first 30 dimensions of the selected 50 dimensions. **(E)** PCA plot of different sample sources after dimensionality reduction. **(F)** Heatmap of the top ten highly variable genes in the first nine dimensions.

Supplementary Figure 2 Heatmap of EPCs cell CNV (Copy Number Variation) status.
