# Supplementary figures and images for: Deciphering the molecular landscape: integrating single-cell transcriptomics to unravel myofibroblast dynamics and therapeutic targets in clear cell renal cell carcinomas
Source: Front Immunol. 2024 Mar 18;15:1374931. doi: 10.3389/fimmu.2024.1374931 (PMC10982338; doi:10.3389/fimmu.2024.1374931)

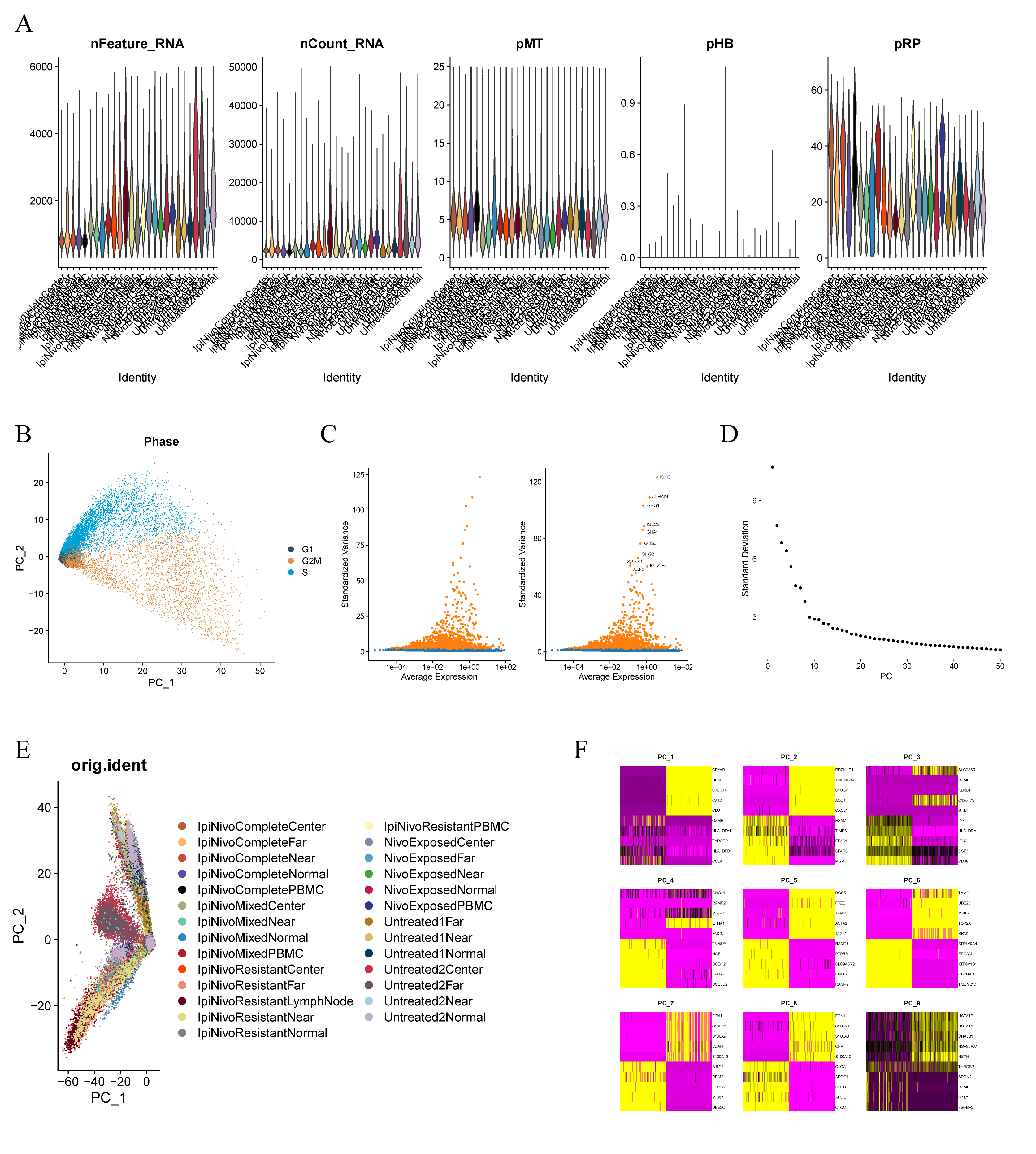

Supplement: Supplementary Figure 1 — Quality control and dimensionality reduction clustering of data. (A) Quality-controlled and filtered single-cell data of ccRCCs. (B) Filtered ccRCCs cell stage examination showing G1, G2M, and S stages. (C) Selection of the top 2000 highly variable genes based on gene expression and dispersion, with the top 10 highly variable genes indicated. (D) RunPCA dimensionality reduction plot showing the first 30 dimensions of the selected 50 dimensions. (E) PCA plot of different sample sources after dimensionality reduction. (F) Heatmap of the top ten highly variable genes in the first nine dimensions. [file Image_1.tif]

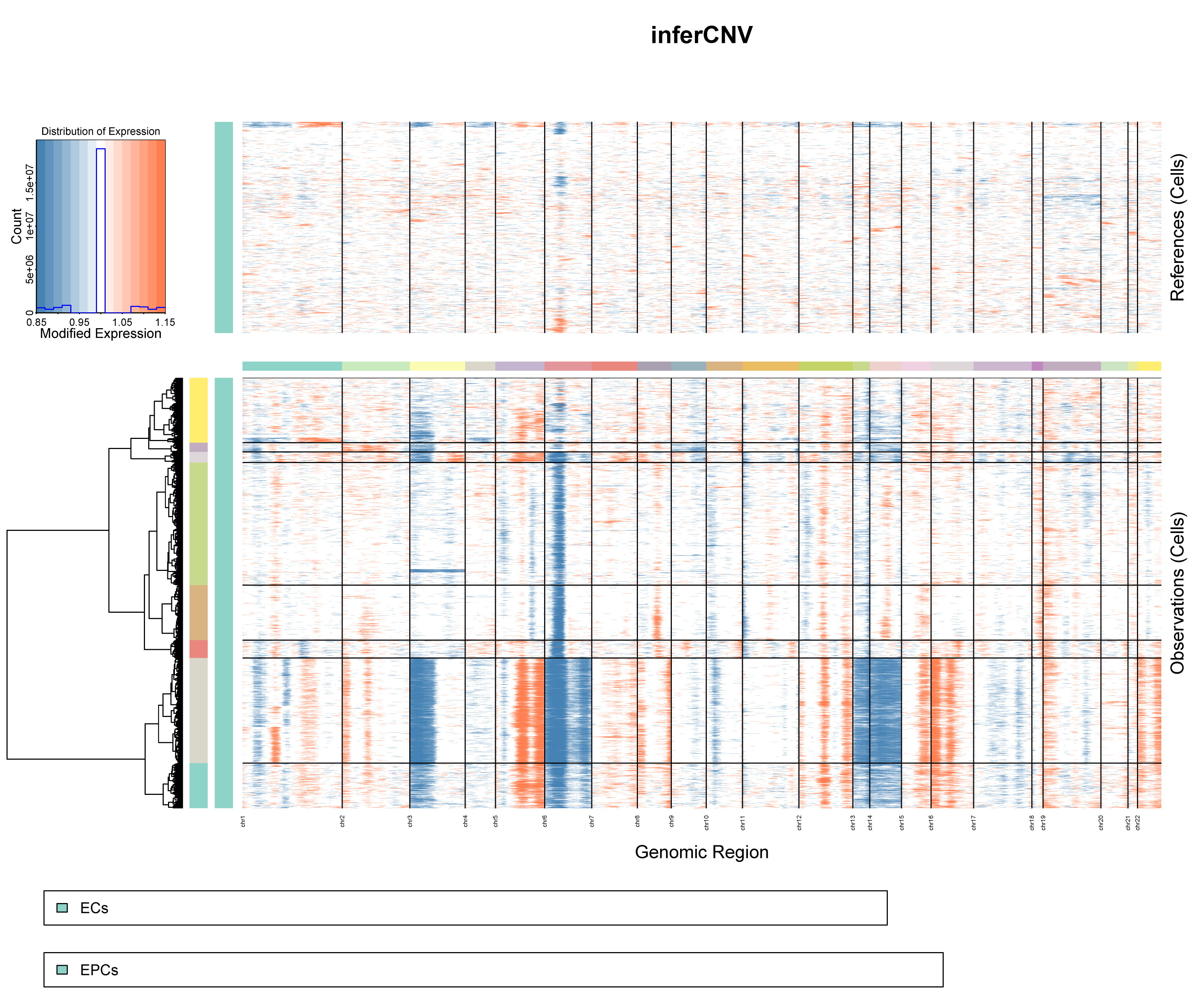

Supplement: Supplementary Figure 2 — Heatmap of EPCs cell CNV (Copy Number Variation) status. [file Image_2.tif]
